# The development of a phosphite-mediated fertilization and weed control system for rice

**DOI:** 10.1038/srep24941

**Published:** 2016-04-25

**Authors:** Mrinalini Manna, V. Mohan M. Achary, Tahmina Islam, Pawan K. Agrawal, Malireddy K. Reddy

**Affiliations:** 1 Plant Molecular Biology Group, International Centre for Genetic Engineering and Biotechnology, Aruna Asaf Ali Marg, New Delhi-110 067, India; 2 Department of Botany, University of Dhaka, Dhaka-1000, Bangladesh; 3 National Agricultural Science Fund, Indian Council of Agricultural Research, Krishi Anusandhan Bhawan-1, Pusa, New Delhi-110 012, India

## Abstract

Fertilizers and herbicides are two vital components of modern agriculture. The imminent danger of phosphate reserve depletion and multiple herbicide tolerance casts doubt on agricultural sustainability in the future. Phosphite, a reduced form of phosphorus, has been proposed as an alternative fertilizer and herbicide that would address the above problems to a considerable extent. To assess the suitability of a phosphite-based fertilization and weed control system for rice, we engineered rice plants with a codon-optimized *ptxD* gene from *Pseudomonas stutzeri*. Ectopic expression of this gene led to improved root growth, physiology and overall phenotype in addition to normal yield in transgenic plants in the presence of phosphite. Phosphite functioned as a translocative, non-selective, pre- and post-emergent herbicide. Phosphite use as a dual fertilizer and herbicide may mitigate the overuse of phosphorus fertilizers and reduce eutrophication and the development of herbicide resistance, which in turn will improve the sustainability of agriculture.

Phosphorus (P) is critical macronutrient that plays a critical role by assimilating into important cellular constituents, such as nucleic acids, phosphoproteins, phospholipids, sugar phosphates, enzymes and energy rich compounds such as adenosine triphosphate (ATP) and nicotinamide adenine dinucleotide phosphate (NADP). P also participates in signaling because phosphorylation and dephosphorylation of target proteins controls diverse cellular functions for proper plant growth and development. The global agricultural demand for this essential plant nutrient is primarily provided by mined rock phosphate (Pi), the only known primary source of P on earth. Nearly 90% of the excavated P is converted into fertilizer. However, P is a non-renewable resource that is speculated to last for a century or two at the current rate of consumption^1^,^2^. To add to this crisis, approximately 80% of applied P fertilizers are lost globally by becoming immobile and unavailable for plant uptake due to absorption, precipitation or conversion into organic forms^3^. To compensate for the low efficiency of P use, excess P fertilizers are routinely applied in crop fields, resulting in not only increased production costs but also environmental degradation via eutrophication^4^. The imminent danger of P scarcity and, inefficient and uneconomical P utilization is further aggravated by crop plant competition with weeds. Weeds are pandemic in their occurrence and account for a significant yield reduction in agriculture. Herbicides are commonly used to control weeds, but in recent years, most weeds have become resistant to the commonly used herbicides^5^.

To address the above-mentioned problems, research to identify suitable solutions, such as alternative strategies for P fertilization, minimization of P losses and overcoming weed problems in crop fields, is urgently needed. Phosphite (Phi or PO_4_^3−^), is a reduced form of P that is readily absorbable by plants through Pi transporters, and its high solubility and low reactivity with soil components make it a suitable source of P. However, plants cannot metabolize Phi, which limits its use as a fertilizer. Phi can only be metabolized naturally by certain bacteria with an enzyme called Phi dehydrogenase (PtxD), which oxidizes Phi into Pi (PO_4_^3−^), a form that can be utilized for various cellular functions^6^,^7^. However, higher life forms, including plants, have evolved to utilize the Pi form of P, thus preventing these organisms from metabolizing Phi because of an absence of cellular processes for Phi oxidation^8^. Several studies have indicated that application of Phi to plants leads to severe growth retardation^9^,^10^,^11^ and might even be lethal, thus making it useful as an herbicide^12^. A recent study by Lopez-Arredondo and Herrera-Estrella reported that genetically engineered tobacco and *Arabidopsis* could be imparted with the ability to metabolize Phi by encoding the *ptxD* gene in their genomes^12^, and Phi was found to function as an herbicide for weeds unable to metabolize Phi.

Successful implementation of a Phi-based fertilization and weed control scheme requires evaluation of this novel technology in crop plants. Therefore, to elucidate the suitability of a Phi-based fertilization and weed control system for rice, an important cereal crop, we generated transgenic rice that encoded a codon-optimized *ptxD* gene from *Pseudomonas stutzeri* and analyzed the Phi metabolism of the transgenic plants. Our studies indicate that *ptxD*-expressing transgenic rice exhibits a potential to harness the benefit of Phi-based fertilization, thus solving the dual problems of a diminishing P reserve and weed management in agriculture.

## Results

### Generation of transgenic rice harbouring a codon-optimized *ptxD*

Because it is well documented that heterologous genes, particularly those of non-plant origin, tend to be expressed poorly in plants, even when driven by a strong promoter, we codon optimized the 1011-bp *ptxD* gene from *Pseudomonas stutzeri* (Supplementary Fig. 1) for the codon bias of the rice plant and chemically synthesized the gene.

To monitor the Phi oxidation properties of the PtxD protein, the codon-optimized *ptxD* gene was over-expressed with the pET28a system, and recombinant PtxD protein was purified to homogeneity using nickel-nitrilotriacetic acid (Ni-NTA)-based affinity chromatography. Recombinant PtxD was visualized on a 12% sodium dodecyl sulfate (SDS) polyacrylamide gel and corresponded to an approximately 37 kDa protein (Supplementary Fig. 2a). Recombinant PtxD effectively oxidized Phi to Pi using nicotinamide adenine dinucleotide (NAD) as a cofactor (Supplementary Fig. 2b), and a native in-gel assay further confirmed Phi mediated NAD hydrate (NADH) production (Supplementary Fig. 2c). Enzyme kinetics data revealed that the V_max_^Phi^ was 0.798 μM/min/μg and the K_m_^Phi^ was 88.46 μmole (Supplementary Fig. 2d). Similarly, the V_max_^NAD^ was 1.146 μM/min/μg, and the K_m_^NAD^ was 146.48 μmole (Supplementary Fig. 2e).

**Figure 1:**
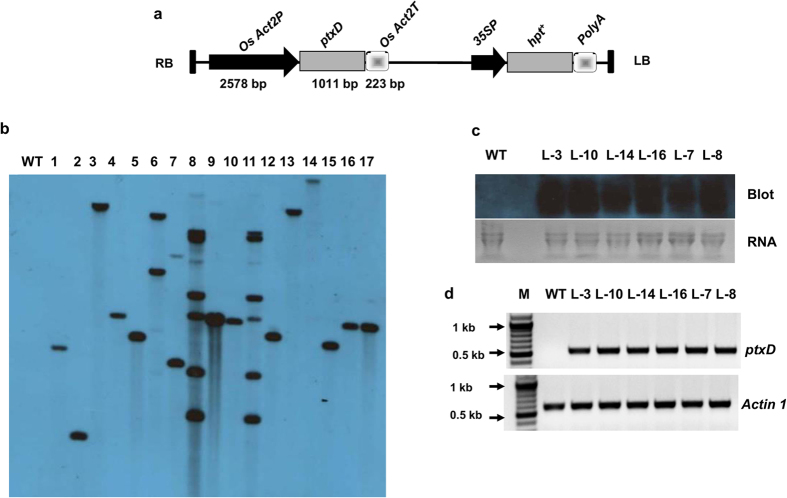
Plant transformation vector details and molecular confirmation of transgene integration and its expression. (**a**) Schematic representation of T-DNA region of plant transformation vector (pMDC99) showing the *ptxD* gene expression cassette consisting of Right Border (RB), rice Actin2 promoter (*Os Act2P*), phosphite dehydrogenase gene (*ptxD*), rice Actin2 terminator (*Os Act2T*), 35S promoter (35SP), hygromycin phosphotransferase gene (*hpt*^+^), PolyA (*PolyA*) and LB Left Border (LB). (**b**) Southern blot showing *Sac* II digested genomic DNA from wild type (WT) and transgenic rice lines (1–17) probed with DIG labeled *hpt* gene. (**c**) Northern blot revealing the *ptxD* transcript from of T_1_ transgenic and WT plants. (**d**) Semi quantitative RT-PCR revealing expression of *ptxD* and *Actin1* gene in transgenic and WT plants. M indicates 100 bp plus DNA ladder.

The *ptxD* expression cassette was cloned under the regulation of the rice Actin2 promoter (*OsAct2P*) and corresponding Actin2 terminator (*OsAct2T*) and subsequently inserted into pMDC99 vector for rice transformation (Fig. 1a). The rice Actin2 constitutive promoter was chosen to achieve a high level of transgene expression for easy oxidation of Phi throughout the plant after absorption by Pi transporters. We generated the *ptxD*-overexpressing transgenic rice in Nipponbare variety of Japonica rice using *Agrobacterium tumefaciens* mediated transformation. The hygromycin selected T_1_ transgenic plants were confirmed by polymerase chain reaction (PCR; Supplementary Fig. 3), and Southern analysis was performed to confirm the copy number of transfer deoxyribonucleic acid (T-DNA) insertions using the hygromycin phosphotransferase (*hpt*) gene as a specific probe (Fig. 1b). We obtained 7 single-copy, one double-copy and 2 multi-copy (containing 5 or 9 copy insertions) transgenic lines. The expression of *ptxD* was confirmed by Northern analysis (Fig. 1c) and semi-quantitative reverse transcription PCR (RT-PCR; Fig. 1d) in 4 single-copy, the double-copy and the 9-copy lines. We did not observe any significant difference in expression levels among the single- and multi-copy insertion transgenic lines. However, we chose single-copy integrated transgenic lines from the T_2_ generation for physiological analysis.

### *ptxD* transgenic plants grow with improved root development and chlorophyll retention in Phi-containing media

**Figure 2:**
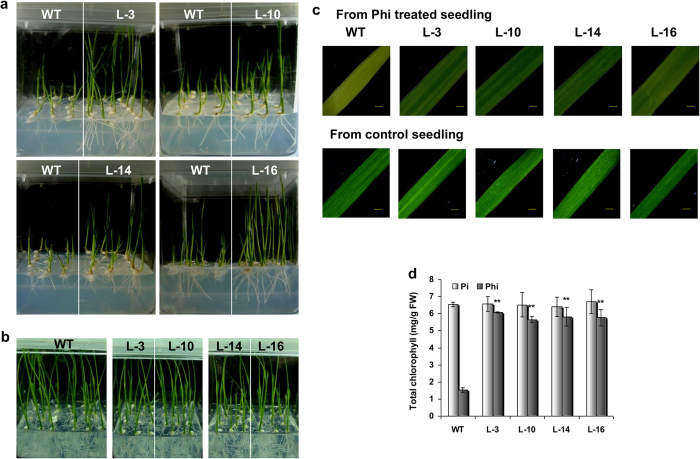
Comparison of effect of Phi on seed germination, seedling morphology and chlorophyll content of WT and transgenic rice. (**a**) Comparison of seedling and root growth morphology of T_2_ *ptxD* lines and WT rice grown in half strength MS media supplemented with 25 mM Phi. (**b**) Photograph showing seedling morphology of transgenic and WT plants grown without Phi supplementation in half strength MS media. (**c**) Leaf blade phenotype of WT and *ptxD* lines showing darker-green leaves surface in transgenic plants under Phi (25 mM) treatment; scale bars representing 10 μm. (**d**) Comparison of chlorophyll content in transgenic lines and WT plants with or without Phi treatment. Data represent mean of independent isolations ± SEM (n = 6).

To validate the ability of the transgenic rice to grow in the presence of Phi, seeds from both wild-type (WT) and transgenic plants were germinated on half-strength Murashige and Skoog (MS) media supplemented without and with 25-mM Phi respectively. Both WT and transgenic seedlings grew well in the absence of Phi (Fig. 2b). However, the transgenic lines exhibited improved growth, proper root development (Fig. 2a) and greater chlorophyll retention (Fig. 2c,d) in comparison to the WT plants under the same Phi treatment conditions, thus indicating that the transgenic plants that overexpressed PtxD were unaffected by the growth inhibition effect of Phi and could utilize Phi as a P source.

### Physiological characterization of transgenic plants during sustained Phi treatment

**Figure 3:**
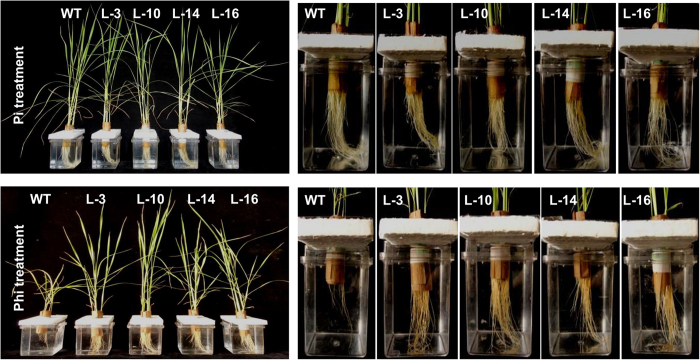
Seedling and root morphology of WT and *ptxD* lines. Comparative growth and root development of WT and T_2_ *ptxD* transgenic lines under Pi or Phi treatment in hydroponic solution.

**Figure 4:**
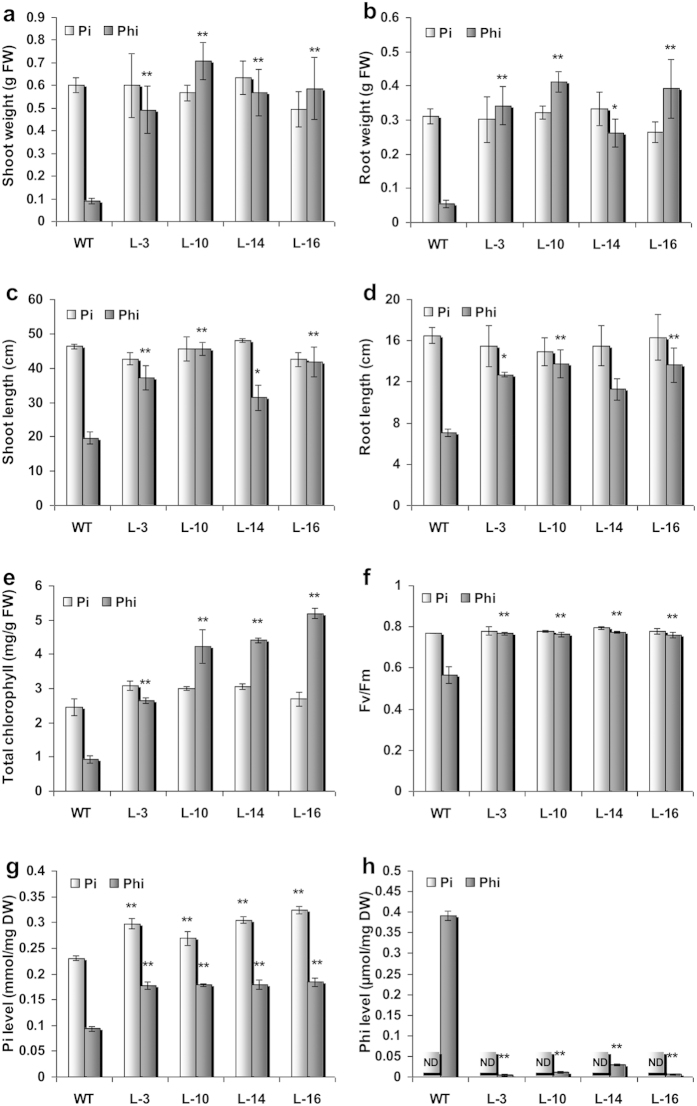
Comparison of physiological parameters of WT and *ptxD* lines under Pi/Phi treatment. WT and T_2_ *ptxD* lines were grown in hydroponic solution for 65 days containing either Pi or Phi. Phi treatment significantly increased *p* ≤ 0.05 (*) or p ≤ 0.01 (**) shoot weight (**a**), root weight (**b**), shoot length (**c**), root length (**d**), total chlorophyll content (**e**), photosystem II activity; Fv/Fm (**f**), in *ptxD* transgenic lines compared to WT plants. (**g**) Either Pi or Phi individual treatment significantly increased the (Pi) level in *ptxD* lines compared to WT plants. (**h**) However, WT plant accumulated more Phi compared to *ptxD* lines under Phi treatment. Each bar represents mean of five independent isolations ± SEM. ND denotes Phi not detected.

To examine the physiology of the transgenic plants during sustained Phi treatment, both WT and transgenic plants were hydroponically grown in Phi-supplemented solutions (Fig. 3) for 65 days, after which various morphological, physiological and biochemical parameters were measured. Plants were simultaneously grown in the presence of Pi as a control (Fig. 3). Phi treatment resulted in very poor root development and severe growth retardation in WT plants compared with those grown in the presence of Pi (Fig. 3). However, transgenic plants under sustained Phi treatment grew as healthily as Pi-supplemented plants, and significant increases in shoot and root biomass (Fig. 4a,b), and shoot and root length (Fig. 4c,d) in the transgenic plants compared with the WT plants were observed. Additionally, the transgenic plants accumulated significantly higher chlorophyll levels than the WT plants (Fig. 4e) and had improved photosystem II (PS II) activity (Fig. 4f). The Pi concentration in both the Pi and Phi treated plants revealed that there was a significant (*p* ≤ 0.01) increase in Pi levels in the transgenic plants compared with the Pi or Phi-treated WT plants (Fig. 4g). Spectrophotometric determination of the Phi level revealed almost no trace of Phi residue in the transgenic plants that were grown in the presence of Phi for 65 days (Fig. 4h). In contrast, WT plants accumulated very high levels of Phi after similar Phi treatment conditions. These results suggest that PtxD-expressing transgenic plants effectively metabolize Phi and grow vigorously with improved biomass and photosynthetic efficiency in a Phi-rich environment.

### Assessing plant growth under different levels of Phi supplementation

**Figure 5:**
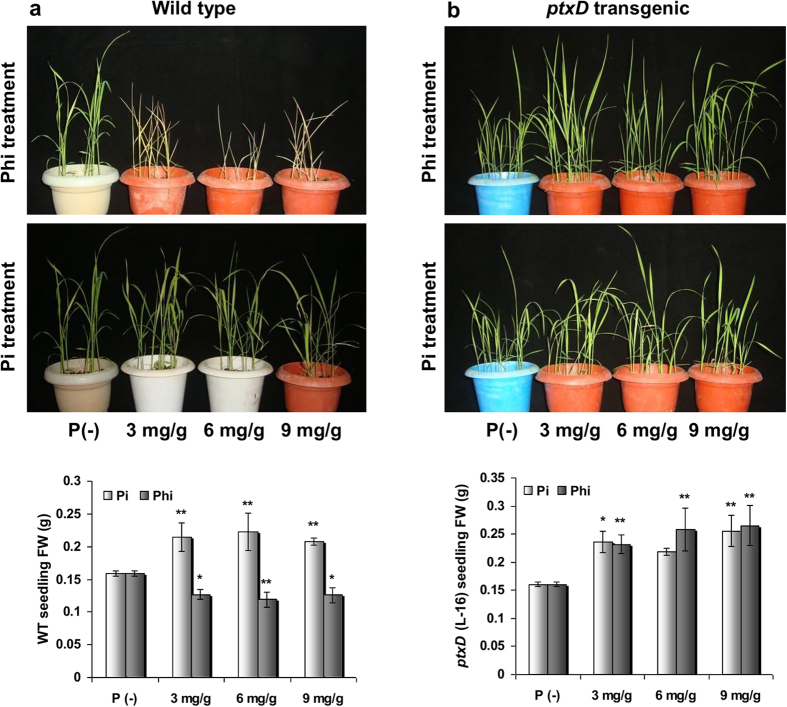
Influence of different levels of Pi or Phi on biomass accumulation in WT and transgenic seedlings. (**a**) Phi treatment at any given level resulted in death of WT seedling after 15 days of application. On the contrary, the biomass of WT seedling increased *p* ≤ 0.01 (**) significantly upon Pi supplementation. (**b**) Both Pi and Phi treatment at all the given levels significantly increased *p* ≤ 0.05 (*) or 0.01 (**) the biomass accumulation in *ptxD* transgenic plant (L-16). Each bar represents mean of 15 independent seedling ± SEM.

To assess and compare biomass accumulation, both WT and transgenic plants were grown in sterile vermiculite with increasing Phi concentrations. Plants grown with a similar supplementation of Pi served as controls. P deprivation resulted in significantly less biomass production in both WT and transgenic plants, and Pi supplementation irrespective of application level resulted in significantly greater biomass production both in WT and transgenic plants compared with the corresponding controls grown in the absence of Pi (Fig. 5a,b). Phi supplementation at every level tested caused the death of the WT rice seedlings within 15 days of sowing (Fig. 5a). In contrast, transgenic rice seedlings generated similar biomass amounts irrespective of the Phi supplementation level (Fig. 5b), thus indicating that Phi supplementation increased the growth and vigor of transgenic plants similarly to Pi at all of the doses tested in this study (Fig. 5a,b). This result also suggests that lower levels of Phi supplementation will not result in a growth penalty for the transgenic plants.

### Phi has potential use as a pre-emergent herbicide

**Figure 6:**
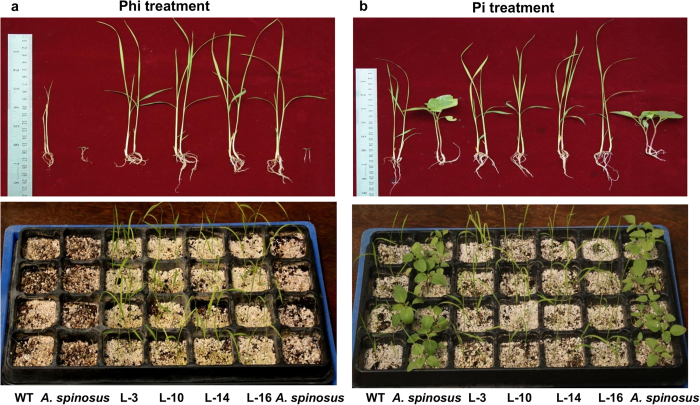
Analysis of pre-emergent herbicidal action of Phi on plant growth. (**a**) Growth and development of WT rice, weed (*Amaranthus spinosus*) and transgenic plants under Phi treatment; observation recorded on 14^th^ day after treatment. Phi treatment resulted in death of WT rice seedlings whereas transgenic plants (L-3, L-10, L-14 and L-16) grew as healthy as Pi supplemented one. Seedlings of *Amaranthus spinosus* had inconspicuous growth. (**b**) Comparative morphology of WT rice, weed and transgenic plants under Pi treatment; observation recorded on 14^th^ day after treatment. All the plants grew healthily in response to Pi treatment.

**Figure 7:**
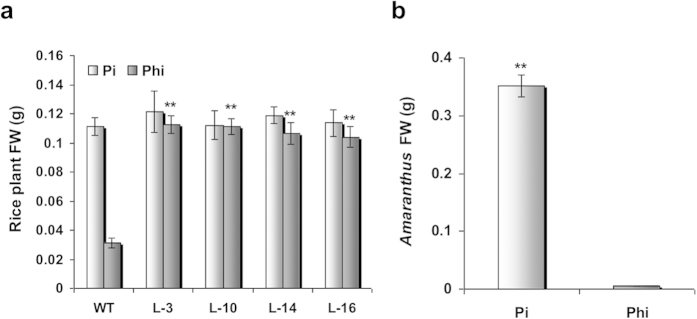
Comparison of biomass accumulation in plants under Pi or Phi treatment. (**a**) The histogram representing total biomass production in WT and transgenic rice lines (L-3, L-10, L-14 and L-16) in response to Pi or Phi treatment. **(b)** The histogram representing total biomass production in *A. spinosus* grown under Pi or Phi supplementation. The graphs indicate mean fresh weight of 8 seedlings ± SEM.

To test the feasibility of Phi as a pre-emergent herbicide, we grew transgenic rice, WT plants and the weed *Amaranthus spinosus* in sterile soilrite supplied either with Phi or Pi (Fig. 6a,b). The WT rice plants died within two weeks of Phi application (Fig. 6a), and most of the *Amaranthus* seeds either failed to germinate or exhibited inconspicuous growth (Fig. 6a). However, Phi application to *ptxD*-expressing transgenic rice resulted in improved growth of the seedlings (Fig. 6a). Pi supplementation aided the vigorous growth of WT rice, transgenic rice and *Amaranthus* (Fig. 6b). Consequently, Phi-treated transgenic plants accumulated biomass comparable to Pi-treated transgenic plants and significantly greater biomass compared to Phi-treated WT rice (Fig. 7a). Phi treatment also resulted in significantly decreased biomass for *Amaranthus* compared with Pi-treated weeds (Fig. 7b), thereby confirming the potential of Phi as a pre-emergent herbicide to kill or suppress weeds in crop fields.

### Phi can be used as a post-emergent herbicide

**Figure 8:**
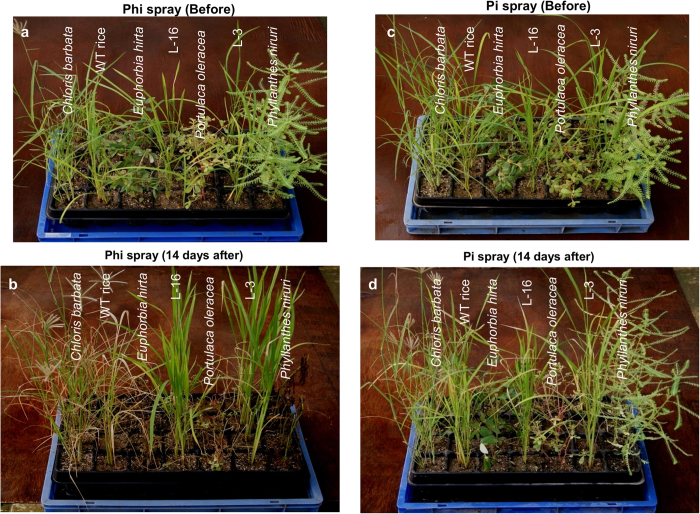
Analysis of post-emergent herbicidal action of Phi. (**a,b**) The figures represent the post emergent herbicidal action of Phi before and after foliar application on weeds, WT plant and transgenic rice lines. (**c,d**) Pi application on weeds, WT plant and transgenic rice lines did not result growth penalty upon foliar treatment.

Because Phi application to soil might raise concerns regarding the potential impact of this toxic chemical on soil microflora or the potential oxidation of Phi by soil microbes, thus limiting the efficiency of this technology or reducing the effectiveness of Phi on weeds because of the presence of a Pi reserve in soil, we analyzed the efficacy of foliar-based Phi application as a weed control system. To determine a lethal dose of Phi, we applied different concentrations of Phi to *Oxalis*, one of the common weeds that infest rice fields (Supplementary Fig. 4). Foliar application of Phi (50, 100 and 250 mM) to *Oxalis* resulted in the scorching of leaf margins on day 14 of Phi spray and, ≥500 mM resulted in drying of the whole plant (Supplementary Fig. 4). Next, we tested foliar application of Phi (500 mM) as a post-emergent herbicide on four different rice field weeds, growing them with WT and transgenic rice lines (Fig. 8a). Phi spray resulted in growth inhibition and severe injury to the WT plants, whereas *ptxD*-expressing lines grew vigorously. Phi application killed two broadleaved weeds, *Phyllanthes niruri* and *Euphorbia hirta*, but caused only growth retardation in *Portulaca oleracea.* The leaves of the monocot weed *Chloris barbata* were bleached, and the plants appeared unhealthy (Fig. 8b). Moreover, upon closely analyzing the leaves of the WT rice, we observed that the leaf tips were initially bleached, after which a chlorosis pattern spread over the whole leaf surface (Supplementary Fig. 6), an important observation indicates that Phi translocated inside the plant. It is important to note that WT rice, transgenic rice and weeds grew vigorously when sprayed with 500 mM of Pi (Fig. 8b). In this weed competition study, it was confirmed that Phi can be used as a post-emergent herbicide to non-specifically kill a variety of weeds with variable life cycles.

### *ptxD*-expressing transgenics rice maintains normal growth and yield when sprayed with Phi

To evaluate the effect of Phi on the yield potential of transgenic and WT plants, we sprayed 500-mM Phi on one-month old rice seedlings three times and, then allowed growth until maturity. The WT plants were unable to survive upon treatment, whereas both WT and transgenic plants grew normally under control condition (Supplementary Fig. 5). To assess the relative yield potential, different parameters such as the plant height, number of panicles per plant, panicle length, spikelet number, seed set rate, weight per 100 grains and average yield were measured and compared for both WT and transgenic plants under control and Phi-treated conditions (Table 1). This analysis revealed that there was no significant variation in the yield parameters from Phi-sprayed transgenic rice plants.

## Discussion

There is widespread concern that the Pi rock reserve will be exhausted in the near future if its current consumption rate is maintained and the efficiency of Pi-based fertilization is not improved^5^. Additionally, herbicide-resistant weeds are likely to jeopardize agricultural productivity by competing with crop plants for scarce soil resources, including water and nutrients. Phi, a reduced form of P, might solve both these problems, because not only can it be utilized to control weeds but it can also be used as a fertilizer in crop plants engineered to express bacterial Phi dehydrogenase, which imparts Phi-metabolizing characteristics to the transgenic plants. Here, we report promising results for Phi-based weed control and fertilization in rice crops.

Phi-metabolizing transgenic plants exhibited comparable physiological fitness when grown in Phi as the sole P source to Pi-grown plants in terms of overall growth, root proliferation, chlorophyll accumulation, PS-II activity and Phi and Pi accumulation. Previous studies have reported Phi mediated growth retardation in a wide variety of plant species^9^,^10^,^11^,^12^,^13^,^14^,^15^,^16^. For instance, root growth of onion (*Allium cepa*)^13^ and *Brassica nigra*^14^ were severely restricted by application of Phi. In spinach, shoot dry weight decreased in response to decreasing phosphate:phosphite ratios^10^. Similar results were seen in case of *Brassica napus* var Peruviridis^16^. In sweet potato, there was decrease in growth velocity, length and dry weight of plants in response to increased treatments of Phi^11^. Our previous work indicated that increasing concentrations of Phi in media led to progressive decrease in shoot and root biomass, root hair formation and chlorophyll accumulation in rice^17^. Similar results were witnessed by Ticconi *et al*.^9^ in *Arabidosis*. Interestingly, Lopez-Arredondo and Herrera-Estrella^12^ discovered that Phi metabolism by transgenic *Arabidopsis* and tobacco plants expressing bacterial PtxD protein generated an improved phenotype and better physiology in terms of greater biomass production, yield and lesser Phi accumulation. Our results revealed that when rice plants acquired the capacity to oxidize Phi, there was marked improvement in the growth, phenotype and physiology of the rice seedlings with improved Pi accumulation and lesser Phi accumulation compared to WT rice plants subjected to similar Phi treatment. Hence, Phi can suitably be substituted for Pi as a source of P in the transgenic plants.

There might be more than one possibility of how Phi compromise growth and development of plants. Most importantly, Phi cannot be oxidised into Pi by the plants as they do not process any enzyme or cellular mechanism to do so. Consequently, the cellular machinery fails to use and metabolize Phi due to their strict specificity towards Pi ion. This might severely impair various key metabolic processes resulting in Phi mediated growth inhibition of plants. The second possibility is the ability of Phi to attenuate the Pi starvation machinery of the cell^9^. Attenuation of Phosphate Starvation Responses (PSRs) leads to failure of plant to sense the deficiency of Pi in Phi rich environment as presence of structurally analogous Phi is perceived as presence of Pi by the plants^9^. Due to this, plants do not induce Pi scavenging machinery which enables plants to acquire P in case of P deficiency.

To meet the food requirements of an ever-growing population, a massive amount of P has been unearthed and applied to crop fields as fertilizer. Unfortunately, nearly 80% of this non-renewable resource is washed away into the ocean, and once, P becomes part of the oceanic water, its concentration is so dilute that it becomes nearly impossible to recycle^18^. Furthermore, marine sediments containing P are not easily accessible, raising doubt regarding the sustainability of crop production in the face of accelerating P depletion in nature. We observed that transgenic seedlings produced greater biomass when fertilized with Phi compared with Pi-starved seedlings, and there was no reduction in biomass production irrespective of the level of Phi supplementation, thereby indicating that there is potential to economize the use of the scarce resource P in the ecosystem. Lopez-Arredondo, D. L. and Herrera-Estrella, L.^12^ found that under greenhouse conditions, transgenic *Arabidopsis* and tobacco plants expressing PtxD protein utilized 30–50% less P when supplemented with Phi to attain similar productivity to that obtained by the same plants using Pi based fertilizer. Hence, there is a possibility to economize P use in the actual field conditions. In this respect, it is important to mention that farmers tend to apply more Pi fertilizer than is actually required because the majority of applied Pi is converted into insoluble inorganic forms or unavailable organic forms by soil microbes^12^. Using Phi-based fertilizer would be advantageous in this regard because it has high solubility and lower reactivity with cations present in soil. Furthermore, microorganism-mediated conversion of Phi into unavailable organic forms can be largely prevented because the majority of soil microbes cannot metabolize Phi. These properties of Phi could economize the use of fertilizer. It is known that in several parts of the world, agricultural soils are in low- or medium-P-fertility categories and do not have sufficient levels to meet the P demands of current high-yielding crop varieties. Phi-based fertilization and weed control could be applied in pockets of land where there is a scarcity of Pi.

We found that Phi can serve as both a pre- and post-emergent herbicide and demonstrated that it controlled both monocot (here WT rice, *Chloris barbata*) and dicot (here *Oxalis, Phyllanthes niruri, Euphorbia hirta, Portulaca oleracea* and *Amaranthus spinosus*) plants effectively. An earlier study by Lopez-Arredondo and Herrera-Estella^12^ also found that Phi effectively controlled a wide variety of both monocot and dicot weeds. It is very important to note that the post-emergent application of Phi can be mediated via fertigation, in which Phi would be applied to plants through irrigation water, thus reducing the burden of separately applying herbicide as required for conventional herbicides. Moreover, foliar application decreases the chance of Phi reaching the soil and subsequent loss into bodies of water via surface runoff.

In recent years, the persistent use of herbicides in agriculture has led to the evolution of a large number of herbicide-resistant super weeds, which has further necessitated constant research in search of new molecules with new targets for herbicidal actions^19^. Because conventional herbicides target specific enzymes, the herbicide molecules bind to the enzyme’s catalytic site, just a few mutations in the active site can significantly reduce the herbicide binding to its target site, thus resulting in the rapid evolution of herbicide-resistant weeds^12^. Constant use of the same herbicide further aids the evolution of these weeds. Other mechanisms that generate herbicide tolerance in weeds are the prevention of herbicide uptake or sequestration of the herbicides in subcellular compartments. Because Phi is structurally analogous to the Pi ion, it is absorbed by plants via Pi transporters^20^, thus making it difficult for plants to prevent its uptake. Moreover, because of its structural analogy with the Pi ion, it might have multiple targets of action inside the cell. Therefore, multiple target sites inside the cell would need to be altered to prevent interaction with Phi, which would be energetically costly for the cell and quite unfeasible. The only mechanism by which weeds can acquire resistance to Phi is by evolving the ability to metabolize or oxidize Phi, which would require the appearance of a new gene in the weed genome, which is impossible in such a short span of time^12^. For these reasons, the use of Phi as an herbicide might decelerate the pace of evolution of herbicide-tolerant weeds in nature even if it does not halt them entirely.

The excessive use of Pi along with nitrogenous fertilizers causes pollution of aquatic bodies, where the occurrences of algal blooms and dead zones are major problems^21^. Even Pi concentrations in the micromolar range promote massive proliferation of algae in the ocean that results in a depletion of aquatic oxygen, leading to the death of aquatic flora and fauna^21^. However, Phi is not metabolized by algae^22^, which will not result in algal blooms even if Phi enters the aquatic systems. Moreover, because Phi is not metabolized by higher-order organisms, it would not create a negative impact on biodiversity. Although Phi is not utilized by the majority of microorganisms, studies have demonstrated slow conversion of Phi into bioavailable Pi by soil microorganisms^23^, thus indicating that this toxic chemical will not accumulate in the soil or water, which could lead to biosafety issues.

To conclude, a system of agricultural production involving cultivation of *ptxD*-expressing transgenic crops that utilizes Phi as a dual fertilizer and herbicide, has a potential as an economical and effective agricultural practice that would enable us to grow crops in soils with low orthophosphate availability and, address the problems of P depletion and herbicide resistance, in addition to mitigating excessive P use to a considerable extent.

## Methods

### Chemical synthesis of *ptxD* gene, purification of recombinant protein and enzyme kinetics

The *ptxD* gene was codon optimized for rice and was chemically synthesized (Life Technologies, USA). *E. coli* BL21 (DE3), harbouring the pET28a_*ptxD* plasmid was grown in liquid LB medium till OD_600_ reached about 0.5 and was subsequently induced with 1 mM isopropyl 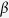-D-1-thiogalactopyranoside (IPTG) for overnight at 18°C. Recombinant PtxD protein was purified by Ni-NTA affinity chromatography. Phi oxidation assay and in-gel assay for the purified recombinant protein were performed according to Costas *et al*.^7^. The molar absorption coefficient of NADH at 340 nm is 6,220 M^−1^cm^−1^ and was used to calculate the specific activity of PtxD. Enzyme kinetic parameters of PtxD were determined by measuring activity at varying concentration of substrate and cofactor NAD. Different enzyme kinetics parameters were calculated by extrapolating Hanes–Woolf plot.

### Construction of plant expression cassette of *ptxD*

Rice *Act2* promoter and *Act2* terminator were PCR amplified from genomic DNA of Nipponbare variety of Japonica rice and cloned in gateway compatible Entry Vector 1. The *ptxD* gene was subsequently cloned in Entry Vector 1 in between the *Act2* promoter and *Act2* terminator. The complete gene cassette was transferred into the plant transformation vector (pMDC99; containing *hpt* gene as plant selection marker) by using LR recombinase mediated gateway cloning process.

### Generation and screening of transgenic plants

The *ptxD* expression cassette (pMDC99- *OsAct2* P: *ptxD: OsAct2* T) was transformed into Nipponbare cultivar of Japonica rice through *Agrobacterium* mediated transformation. Fifteen-day-old embryogenic calli were infected with EHA105 *Agrobacterium* strain harbouring the pMDC99 vector and co-cultivated for 48 h on N6 medium supplemented with acetosyringone. The transformed cells were selected on N6 medium supplemented with 50 mg/L hygromycin. Secondary calli or microcalli that developed after three rounds of selection were transferred to regeneration media for development of green shoots. The regenerated plantlets were transferred in rooting media to facilitate rooting. The putative independent transgenic plants were transferred to vermiculite for hardening followed by transferring to soil pots in the greenhouse. T_1_ seeds were screened by geminating them on 30 mg/L hygromycin containing media followed by PCR confirmation using *ptxD* gene specific primers (*ptxD* F: ACATTGCGAGCTGATGACCA and *ptxD* R: TGAAATCGGAGGAGGCGAAC).

### Molecular conformation of transgenic plants

For Southern blot hybridization analysis, 20 μg total DNA from wild type rice and 17 T_1_ transgenic lines was separately digested with 50 U of *Sac* II restriction enzyme (New England Biolabs). Restriction digested genomic DNA fragments were size-fractionated by electrophoresis on a 0.8% agarose gel and subsequently transferred onto N^+^ nylon membrane. The blot was hybridized with 1 kb *hpt* specific fragment that was prepared by PCR DIG Probe synthesis kit (Roche, Germany). Hybridization and post-hybridization washing conditions and detection of signals were performed according to instructions provided in the kit (Roche, Germany).

For Northern blot analysis, total RNA from 30-d-old transgenic and WT plants was isolated using Trizol reagent. 20 μg of the total RNA was run on denaturing agarose gel containing formaldehyde and then transferred to a N^+^ nylon membrane. The blot was hybridized with *ptxD* coding region (548 bp amplified using above primers) as probe and further steps were performed as per manufactures instruction (Roche, Germany). For semi-quantitative RT-PCR analysis, first-strand cDNA was synthesized from 5 μg of total RNA using oligo (dT) primer using SuperScript III (Invitrogen). Semi quantitative RT-PCR was performed using this cDNA as template. Equal amount of cDNA as template in each PCR reaction was ensured by using house-keeping gene rice actin 1 (*Actin 1*) as the standard in PCR (for selecting *Actin 1* as reference gene, we checked the expression pattern of five housekeeping genes of rice namely *Actin 1, Tubulin, Ubiquitin 5, 18S rRNA* and *eIF-1α* across six transgenic lines and WT in leaf tissues. Expression of rice actin gene was either comparable with respect to *Tubulin, 18S rRNA* and *eIF-1α* or better as compared to *Ubiquitin 5*. However, we selected actin 1 gene as the standard in PCR since it has been reported as one of the most stable reference genes in rice^24^). *Actin 1* primer sequences used in the study are as follows: *Actin* F: GCCGTCCTCTCTCTGTATGC and *Actin* R: GCAATGCCAGGGAACATAGT.

### Plant materials and growth conditions

For seedling growth assay, T_2_ transgenic lines and WT rice seed were surface sterilized and were directly grown on half-strength MS medium^25^ in Petri dishes containing or devoid of 30 mg/L hygromycin. The germinated seeds were then placed on 25 mM Phi (Na_2_HPO_3_.5H_2_O) containing media and grown till 14 days in growth chamber under a controlled-environment conditions (25 °C, 12:12 h light/dark cycle).

For hydroponic treatment, one week old transgenic (hygromycin selected) and WT seedlings were grown by placing on wet strips of germination sheet, rolled and finally placed on nutrient solution^26^ containing 0.6 mM of KH_2_PO_4_ or Na_2_HPO_3_.5H_2_O.

For greenhouse experiments, plants were grown in greenhouse under natural light with supplementation from high-pressure sodium bulbs (50 mM m^−2^ s^−1^). Growth condition were provided with a 12-h light (30 °C)/12-h dark (28 °C) photoperiod with approximately 200 μM m^−2 ^s^−1^ photon density and 70% humidity.

Assessment of various doses of Phi on growth of WT and transgenic rice seedlings were carried out by growing them in sterile vermiculite containing 0 mg, 3 mg, 6 mg and 9 mg KH_2_PO_4_ (Pi) or Na_2_HPO_3_.5H_2_O (Phi); 12 mg urea and 6 mg KNO_3_ (per g) respectively. The pots were regularly watered with distilled water and data were recorded 15 days after sowing.

For assessing pre-herbicidal mode of action of Phi, 7 days old WT and transgenic seedlings were planted on pots containing soilrite that were pre supplemented with urea: KH_2_PO_4_ or Na_2_HPO_3_.5H_2_O: KNO_3_ (4:2:2 g per kg of soilrite). At the same time, about 20 seeds of *Amaranthus spinosus* were placed for germination in each hole of the same pots. The pots were regularly watered with distilled water to prevent entry of Pi as far as possible. Data were recorded two weeks after sowing.

To determine the post-emergent herbicidal effect of Phi, 50 ml of solutions containing various concentrations of Na_2_HPO_3_.5H_2_O (10, 50, 100, 250, 500, 750 and 1000 mM strength) were sprayed on *Oxalis* after adding 0.1% of tween20 (to impart surfactant property of the Phi solutions). The results were recorded 7 and 14 days after the spray respectively.

For crop-weed competition experiment, WT plants, transgenic lines and weeds were grown in pots containing soil and vermiculite mixture (2:5) in the green house for 4 weeks. Two plants in each spot were grown in pots and were regularly irrigated with tap water. After 2 weeks, Pi/Phi containing solutions (of 500 mM concentration) containing 0.1% tween20 were sprayed on the seedlings. Each spraying was done with 200 ml of Pi/Phi solutions per tray and it was done thrice with 3 days interval between consecutive sprays. The results were photographed after 14 days after the final spray.

For recording yield parameters, WT and transgenic plants were grown in pots containing soil for one month with regular irrigation with tap water and were subsequently sprayed with or without 50 ml of Na_2_HPO_3_.5H_2_O (500 mM) containing solutions (with 0.1% tween20) per plant thrice at the interval of 3 days between consecutive sprays and further grown till maturity following which various yield parameters were recorded.

### Measurement of physiological and biochemical parameters

Chlorophyll was quantified by method as described by Gould *et al*.^27^. Total chlorophyll was estimated at 663 and 645 nm using following equation [20.2 A_645_ + 8.02 A_663_ X (1/weight of sample in mg)] and expressed in mg/g FW^28^.

PSII activity or F_v_/F_m_ was determined in the third to fifth expanded leaves of WT and transgenic plants grown under Pi and Phi supplemented condition using infra-red gas analyzer (Li-COR 6400-40, Lincoln, NE, USA) with an integrated fluorescence chamber head (Li-COR). Conditions during measurements were photosynthetically active radiation (PAR) of 1000 ± 7 μmol m^−2^s^−1^, relative humidity 79 ± 5%, temperature 30 ± 3 °C and an ambient CO_2_ concentration of 400 μmol mol^−1^. Quantum efficiency of PSII (F_v_/F_m_) was measured in the 30 min dark-adapted leaves, following manufacturer’s settings (Li-COR). Data measured and stored in the machine, was automatically collected by leaf area corresponding to the leaf portion enclosed within the leaf chamber.

For measuring Pi and Phi levels in plants, these ions were initially isolated from leaf samples by water extraction method according to Thao *et al*.^16^. Inorganic Pi was measured by following the protocol of Lanzetta *et al*.^29^ with minor modifications as specified in Manna *et al*.^17^. Inorganic Phi was measured by following the protocol of Manna *et al*.^17^.

### Statistical analysis

The experiments were replicated as indicated below respective figures and pooled data were subjected to one- or two-way analysis of variance (ANOVA) followed by least significance difference (LSD) or honestly significant difference (HSD) test at *p* ≤ 0.05 (*) or 0.01 (**) levels of significance^30^.

## Additional Information

**How to cite this article**: Manna, M. *et al*. The development of a phosphite-mediated fertilization and weed control system for rice. *Sci. Rep.* **6**, 24941; doi: 10.1038/srep24941 (2016).

## Supplementary Material

Supplementary Information

## Figures and Tables

**Table 1:** Growth and yield of WT and *ptxD* transgenic lines under Phi or control conditions.

Plant line		Plant height (cm)	Panicle/Plant	Panicle length (cm)	Seed set rate	Weight per 100 grains (g)	Yield/Plant (g)
WT	Sprayed with Phi	NIL	NIL	NIL	NIL	NIL	NIL
L-3		65.8 ± 3.322	6.6 ± 0.244	16.0 ± 0.447	0.94 ± 0.013	2.21 ± 0.014	11.21 ± 0.518
L-10		65.2 ± 1.984	7.0 ± 0.316	16.6 ± 0.291	0.91 ± 0.014	2.38 ± 0.024	12.32 ± 0.430
L-14		56.6 ± 1.568	5.6 ± 0.244	15.2 ± 0.122	0.95 ± 0.014	2.28 ± 0.033	9.27 ± 0.495
L-16		71.8 ± 2.353	6.2 ± 0.374	15.6 ± 0.244	0.90 ± 0.016	2.18 ± 0.013	10.60 ± 0.712
WT	Control without Phi	60.9 ± 1.615	6.6 ± 0.400	16.0 ± 0.447	0.91 ± 0.010	2.15 ± 0.018	11.26 ± 0.442
L-3		65.0 ± 5.594	6.0 ± 0.447	16.6 ± 0.533	0.92 ± 0.012	2.13 ± 0.001	10.36 ± 0.787
L-10		65.8 ± 3.397	7.2 ± 0.374	15.9 ± 0.640	0.91 ± 0.019	2.13 ± 0.016	11.82 ± 0.794
L-14		66.4 ± 1.364	7.0 ± 0.316	16.8 ± 0.374	0.91 ± 0.006	2.05 ± 0.022	11.23 ± 0.598
L-16		67.3 ± 1.700	6.4 ± 0.244	16.5 ± 0.632	0.90 ± 0.014	2.33 ± 0.015	11.25 ± 0.350
