# Supplementary material for: The development of a phosphite-mediated fertilization and weed control system for rice
Source: Sci Rep. 2016 Apr 25;6:24941. doi: 10.1038/srep24941 (PMC4842969; doi:10.1038/srep24941)
Supplement: Supplementary Information [file srep24941-s1.pdf]

SUPPLEMENTARY INFORMATION

The development of a phosphite-mediated fertilization and weed control system for rice

Mrinalini Manna[1], V. Mohan M. Achary[1], Tahmina Islam[1,2], Pawan K. Agrawal[3], Malireddy K. Reddy[1]

[1]Plant Molecular Biology Group, International Centre for Genetic Engineering and Biotechnology, Aruna Asaf Ali Marg, New Delhi-110 067, India.

[2]Department of Botany, University of Dhaka, Dhaka-1000, Bangladesh.

[3]National Agricultural Science Fund, Indian Council of Agricultural Research, Krishi Anusandhan Bhawan-1, Pusa, New Delhi-110 012, India.

Correspondence and requests for materials should be addressed to: MK Reddy (email: reddy@icgeb.res.in)

Tel: +91-11-26741242, Fax: +91-11-26742316

Table of Contents

Supplementary Figure 1. *ptxD* gene sequence used in present study.

Supplementary Figure 2. Overexpression of PtxD enzyme in *E. coli* and its biochemical analysis.

Supplementary Figure 3. PCR confirmation of *ptxD* lines.

Supplementary Figure 4. Determination of lethal concentration of Phi on weed *Oxalis*.

Supplementary Figure 5. Effect of Phi on growth of WT and transgenic rice plants.

Supplementary Figure 6. Visualizing leaf scorching pattern in WT rice in response to Phi spray.

```
ATGCTCCCGAAGCTCGTGATCACCCACAGGGTGCACGATGAGATCCTCCAGCTCCTCGCCCCACA
TTGCGAGCTGATGACCAACCAGACCGACTCTACCCTCACCCGCGAGGAGATTCTCAGGCGCTGCA
GGGATGCCCAGGCCATGATGGCCTTCATGCCAGATAGGGTGGACGCCGATTTCCTCCAGGCCTGC
CCAGAGCTGAGGGTGGTGGGCTGCGCCCTCAAGGGCTTCGATAACTTCGATGTGGATGCCTGCAC
AGCCAGGGGCGTGTGGCTGACCTTCGTGCCAGATCTCCTCACAGTGCCAACAGCCGAGCTGGCCA
TTGGCCTCGCCGTGGGCCTCGGCAGGCATCTCAGGGCCGCCGATGCCTTCGTGAGGTCTGGCGAG
TTCCAGGGCTGGCAGCCACAGTTCTACGGCACCGGCCTCGATAATGCCACCGTGGGCATTCTCGG
CATGGGCGCCATTGGCCTGGCTATGGCCGATAGGCTCCAGGGCTGGGGCGCCACACTCCAGTACC
ATGAGGCCAAGGCCCTCGATACCCAGACAGAGCAGAGGCTCGGCCTCAGGCAAGTCGCCTGCTCT
GAGCTGTTCGCCTCCTCCGATTTCATCCTCCTCGCCCTCCCACTCAACGCCGATACACAGCATCT
CGTGAACGCCGAGCTGCTCGCCCTCGTGAGGCCAGGCGCCCTCCTCGTGAATCCATGCAGGGGCT
CTGTGGTGGATGAGGCCGCCGTGCTCGCCGCTCTTGAGAGAGGCCAGCTGGGCGGCTACGCCGCC
GATGTGTTCGAGATGGAGGATTGGGCCAGGGCGGATAGGCCAAGGCTCATTGATCCAGCGCTCCT
CGCCCATCCGAACACCCTCTTCACACCACATATTGGCTCTGCCGTGAGGGCCGTCAGGCTCGAGA
TTGAGAGGTGCGCCGCCCAGAACATCATTCAGGTGCTCGCCGGCGCCAGGCCAATCAATGCCGCC
AATAGGCTCCCAAAGGCCGAGCCAGCCGCCTGCTGA
```

Figure S1 | *ptxD* gene sequence used in present study. Codon optimized CDS of *ptxD* gene from *Pseudomonas stutzeri* for expression in monocot plant (*O. sativa* L.).

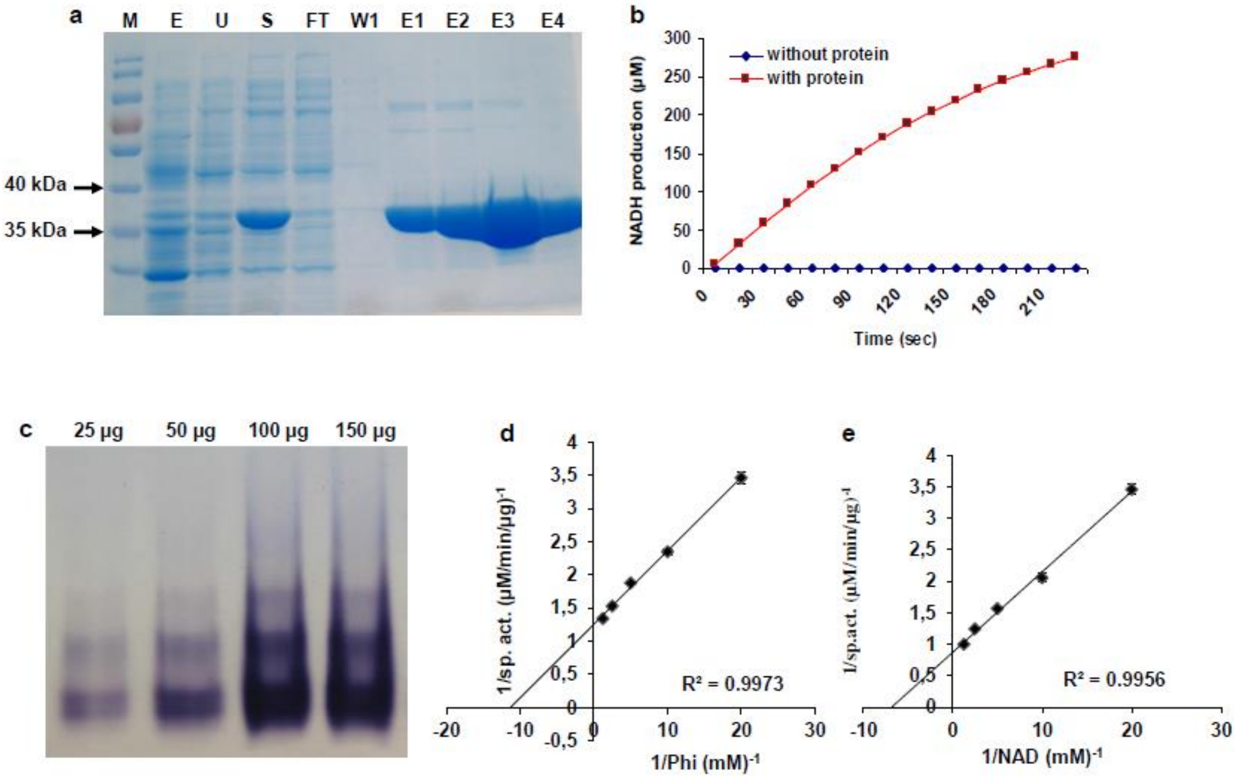

Figure S2 | Overexpression of PtxD enzyme in *E. coli* and its biochemical analysis. (**a**) IPTG induction and purification of recombinant PtxD protein by Ni-NTA chromatography purification. Line M indicates protein marker, E; protein expression in empty pET28a vector, U; uninduced fraction, S; supernatant fraction, FT; flow through, W1; wash 1 and E1 to E4 represent elution fractions. (**b**) Spectrophotometric determination of PtxD activity by monitoring NADH production at 340 nm. (**c**) In-gel assay of PtxD enzyme representing Phi-mediated NADH production. For performing this assay, Native PAGE was carried out at 4 ºC in 12% polyacrylamide continuous gel using 1X Tris-Glycine buffer (pH 8.3) and subsequently, the native PAGE gel slab was incubated for 30 min at 30 ºC in 100 ml of 100 mm Tris, pH 8.5, containing 10 mM Phi, 25 mg of NAD, 30 mg of nitro blue tetrazolium, and 2 mg of phenazine methosulfate as described by Heeb and Gabriel (1984)[31]. Chemical reduction of the nitro blue tetrazolium dye by enzymatically produced NADH resulted in precipitation of dark blue product which could be easily visualized in the stained gel. (**d**) Hanes-Woolf plot depicting relationship between specific activity of PtxD with different Phi concentrations and (**e**) representing relationship between specific activity of PtxD with different NAD concentrations.

*Reference: 31. Heeb, M. J. & Gabriel, O. Enzyme localization in gels. *Methods Enzymol*. **104,** 416–439 (1984).

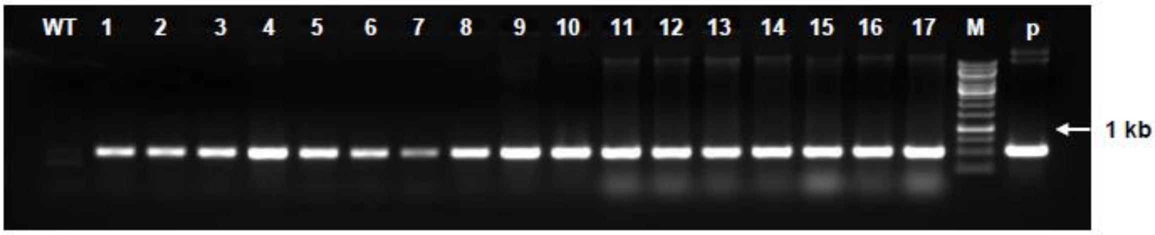

Figure S3 | PCR confirmation of *ptxD* lines. Confirmation of presence of *ptxD* gene in T_1 transgenic rice plants. 1-17 represents 17 transgenic lines, M indicates 1kb plus DNA ladder and p represents plasmid control.

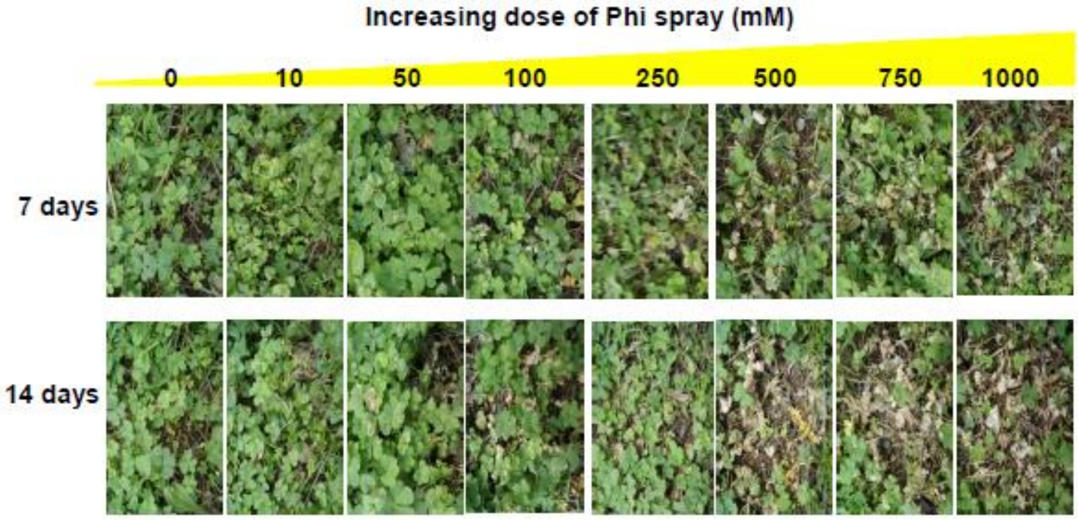

Figure S4 | Determination of lethal concentration of Phi on weed *Oxalis*. Effect of foliar application of Phi (0-1000 mM) on broad leaf weed *Oxalis* 7 and 14 days after the spray. For the foliar application of Phi, 50 ml of solutions containing 0.1% tween20 were spayed in the form of fine mist over the foliar surface of the weeds.

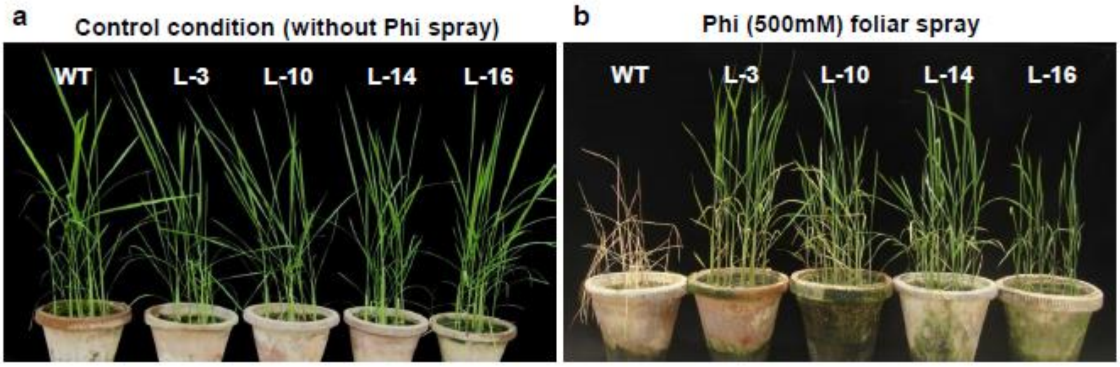

Figure S5 | Effect of Phi on growth of WT and transgenic rice plants. (**a**) Growth and development of WT and transgenic lines under control condition. (**b**) Foliar application of Phi (500 mM) resulted in death of WT plant. On the contrary, *ptxD* transgenic grew fairly healthy similar to control condition. WT and transgenic plants were grown in pots containing soil for one month with regular irrigation with tap water and were subsequently sprayed without or with 50 ml of Phi (500 mM) containing solutions (with 0.1% tween20) per plant thrice at the interval of 3 days between consecutive sprays. Plants were photographed 7 days after the final spray.

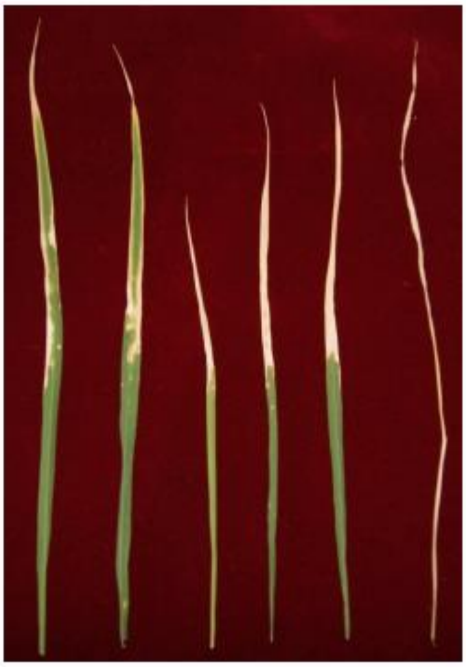

Figure S6 | Visualizing leaf scorching pattern in WT rice in response to Phi spray. Photograph showing leaf bleaching pattern of WT plant starting from the tip and migrating downward.
